# Hyperspectral Imaging (HSI) Technology for the Non-Destructive Freshness Assessment of Pearl Gentian Grouper under Different Storage Conditions

**DOI:** 10.3390/s21020583

**Published:** 2021-01-15

**Authors:** Zhuoyi Chen, Qingping Wang, Hui Zhang, Pengcheng Nie

**Affiliations:** 1 College of Biosystems Engineering and Food Science, Zhejiang University, Hangzhou 310058, China; zhuoyichen@zju.edu.cn (Z.C.); 22013049@zju.edu.cn (Q.W.); 21813051@zju.edu.cn (H.Z.); 2 Key Laboratory of Sensors Sensing, Ministry of Agriculture, Zhejiang University, Hangzhou 310058, China

**Keywords:** hyperspectral imaging, storage conditions, classification, prediction, visualization

## Abstract

This study used visible/near-infrared hyperspectral imaging (HSI) technology combined with chemometric methods to assess the freshness of pearl gentian grouper. The partial least square discrimination analysis (PLS-DA) and competitive adaptive reweighted sampling-PLS-DA (CARS-PLS-DA) models were used to classify fresh, refrigerated, and frozen–thawed fish. The PLS-DA model achieved better classification of fresh, refrigerated, and frozen–thawed fish with the accuracy of 100%, 96.43%, and 96.43%, respectively. Further, the PLS regression (PLSR) and CARS-PLS regression (CARS-PLSR) models were used to predict the storage time of fish under different storage conditions, and the prediction accuracy was assessed using the prediction correlation coefficients (R_p_^2^), root mean squared error of prediction (RMSEP), and residual predictive deviation (RPD). For the prediction of storage time, the CARS-PLS model presented the better result of room temperature (R_p_^2^ = 0.948, RMSEP = 0.255, RPD = 4.380) and refrigeration (R_p_^2^ = 0.9319, RMSEP = 1.188, RPD = 3.857), while the better prediction of freeze was by obtained by the PLSR model (R_p_^2^ = 0.9250, RMSEP = 2.910, RPD = 3.469). Finally, the visualization of storage time based on the PLSR model under different storage conditions were realized. This study confirmed the potential of HSI as a rapid and non-invasive technique to identify fish freshness.

## 1. Introduction

The quality and price of fish depend largely on the freshness [1]. Refrigeration is the main method of short-term storage of fish. During the refrigeration process, fish experienced three stages of rigidity, autolysis, and corruption, which led to a continuous decline in their quality. Freezing is a commonly used effective method for long-term storage of fish [2]. However, during the freezing and thawing process, fish will undergo a series of physiological and biochemical reactions [3], including protein denaturation, fat oxidation, ice crystal formation, tissue damage, enzyme activity changes, and the decomposition of trimethylammonium oxide into trimethylamine, dimethylamine, and formaldehyde, which lead to the gradual deterioration of the nutritional value, texture characteristics, and freshness of fish [4,5].The above process will seriously affect flavor, texture, and color of fish [6], so freezing and thawing will make the sensory and market value low [7]. In order to make more profits, some illegal traders sell frozen–thawed fish as fresh fish. Therefore, it is necessary to study a fast and effective method to identify fresh and frozen–thawed fish and detect the storage time of fish.

At present, microbiological methods are mainly used to predict the remaining storage time of fish [8]. Although the results are reliable, the operation is complicated, and the fish is destroyed and loses its edible value. At the same time, too-long testing time cannot be applied online or on a large scale, nor can it meet the requirements of fast and non-destructive testing. Many scholars at home and abroad used visible/near infrared spectroscopy to detect the storage time of fish. Boknaes et al., (2002), Nilsen et al., (2002), and Ting Wu (2018) used visible/near infrared spectroscopy to detect the storage time of fish and achieved good detection results [9,10,11]. Hyperspectral imaging (HSI) emerges as a non-destructive and rapid analytical tool for assessing food quality, safety, and authenticity [12]. Sivertsen et al., (2011), Kimiya et al., (2013), and Khojastehnazhand et al., (2014) applied HSI technology to detect the refrigerated storage time of fish. Although satisfactory results were obtained, none of them intuitively demonstrated the refrigerated storage prediction time of each pixel on the fish image [13,14,15]. Traditional enzyme analysis and physiological method [16], chemical, microbiological, and sensory methods [17] can effectively identify fresh and frozen–thawed fish, but these methods are detrimental, time-consuming, and require expensive consumables. Physical methods, such as colorimeter, texture analyzer, conductivity meter, electronic nose, etc., can only detect a single parameter related to freshness, which is far from enough to represent the various chemistry, biochemistry, physics, and microbial changes that occur during the freezing–thawing process of fish [10]. Studies have shown that visible/near infrared spectroscopy is feasible for the identification of fresh and frozen–thawed fish [5,18]. The HSI technology that integrates spectral information and image information is a research hotspot in food non-destructive testing. Sivertsen et al., (2011) and Kimiya et al., (2013) applied visible/near-infrared HSI to identify fresh and frozen–thawed cod and Atlantic salmon, respectively [14,15]. Using K-nearest neighbor classifier (KNN) as a classification method, the whole fish fillet was divided into multiple grids, the spectral information of each grid was analyzed, and the whole fish fillet was displayed the correct recognition rate of different spatial locations (different grids) in the form of a pseudo-color map. Junli Xu et al., (2017) used HSI to identify optimum wavelengths carrying most important information to classify between fresh organic, fresh conventional, chill-stored organic, and chill-stored conventional salmon samples [19]. Kathryn E. Washburn et al., (2017) used HSI to realize the identification of fresh, once-thawed, and twice-thawed cod samples, demonstrating that HSI had the potential for use as an online method for evaluation of fish freeze–thaw history [20]. Jiajia Shan et al., (2018) achieved the classification of intact fish with scales, intact scaled fish, skin side of fish fillets, and flesh side of fish fillets with high accuracy [7]. However, few studies have predicted and visualized the storage time of fresh, refrigerated, and frozen–thawed fish simultaneously by using HSI technology.

Therefore, the specific objectives of this study are to (1) analyze the average hyperspectra of different parts of pearl gentian grouper, and select suitable parts for follow-up research; (2) establish partial least square discrimination analysis (PLS-DA) and competitive adaptive reweighted sampling- PLS-DA (CARS-PLS-DA) models to identify fresh, refrigerated, and frozen–thawed pearl gentian grouper; (3) establish PLS regression (PLSR) and CARS-PLSR models to predict the storage time of pearl gentian grouper stored at room temperature, refrigerating and freezing; and (4) visualize the storage time of each pixel of fish under different storage conditions.

## 2. Materials and Methods

### 2.1. Sample Preparation 

The origin of 22 pearl gentian grouper for the study was Wenzhou, China. The pearl gentian grouper was cultured in circulating water, pH 7.5–8.2, culture water temperature 27–30 °C, dissolved oxygen ≥8.0 mg/L. The average weight of pearl gentian grouper with the internal organs removed was 559.0 ± 29.2 g. We removed the head of the fish, washed and dried, cut in half, and the skinless side of fish were prepared for HSI scanning. First, all 22 fresh fish were scanned for HSI immediately (0 day). Then, the fish samples were divided into three groups. One group of 2 fish were stored at room temperature of 10–25 °C for 1, 2, and 3 days until future HSI scanning. Another group of 10 fish were stored at a refrigerator of 4–7 °C for 1, 2, 5, 8, and 13 days. Two refrigerated fish samples were taken out from the refrigerator each time, transferred to a room temperature of 10–25 °C, and left to equilibrate for 1 h for HSI scanning. And the third group of 10 fish were kept in frozen condition of −28 °C for 1, 5, 10, 18, and 30 days until future HSI scanning. Two frozen samples were taken out from the refrigerator each time, thawed in water (room temperature) for 1 h, and left to equilibrate for another 1 h at room temperature before HSI measurement. Fish sample under refrigeration and freezing conditions were not returned to the refrigerator after hyperspectral image were collected.

### 2.2. HSI Equipment

The core components of the HSI system included: Aa high-performance CCD (Charge Coupled Device Camera), a mobile platform for sample movement scanning, an imaging spectrometer (ImspectorV10, Spectral Imaging Ltd., Oulu, Finland) and a computer installed with a data acquisition software. A lighting device was installed above the mobile platform, which contained two 150 W quartz tungsten halogen lamps. The software controlled the entire spectrum acquisition process, including the speed of the mobile platform motor, exposure time, and wavelength range. Spectral images of the prepared samples were acquired in the reflectance mode by employing a laboratory-based line scanning HSI system covering the wavelength region of 900–1700 nm with spectral resolution of approximately 3.37 nm. The acquired three-dimensional hyperspectral images were stored in raw format and then exported to ENVI4.6 software (ITT Visual Information Solutions, Boulder, CO, USA) for subsequent processing.

### 2.3. Data Analysis

#### 2.3.1. Image Acquisition and Calibration

On each sampling day, each grouper sample was placed on the translation stage and then conveyed to the field of view (FOV) camera with a constant speed of 11 mm/s to be scanned line by line. Exposure time of the camera, frame rate, and the motor speed were carefully adjusted to obtain equal pixel resolution of the horizontal and vertical axes and to avoid distortions of images. The image acquisition process was carried out at room temperature.

In order to reduce the influence of the dark current of the camera and the uneven distribution of light source intensity in each band, it is necessary to correct the reflectivity of the original image ($I_{0}$). In the same environment as the sample image collection, the standard white calibration plate (reflectivity close to 100%) was scanned to obtain a white calibration image (W), then the light source was turned off and the lens was covered (reflectivity close to 0%) to collect a black calibration image (B). The corrected images (I) were calculated according to the following formula [21]:

(1) $$I=\frac{I_{0}-B}{W-B}\times 100\%$$

All the corrected images were then used as the basis for subsequent analysis to extract spectral data, important wavelength selection, classification, prediction, and visualization purposes.

#### 2.3.2. Spectral Data Extraction

Background removal is a fundamental step to separate the sample from the background in hyperspectral image, as subsequent extracted data are highly based on the precision of this process [22]. In this study, the remote sensing image processing ENVI4.6 software (ITT Visual Information Solutions, Boulder, CO, USA) was used for selecting the Region of Interest (ROI) that did not contain background information for the corrected hyperspectral image and extract ROIs for each sample. The reflectance spectra of all pixels within the ROIs were averaged to represent each part of sample. Then Matlab9.4 R2018a software (The Mathworks Inc., Natick, MA, USA) was used for subsequent processing. The collected hyperspectral images have a total of 256 bands, and the first 19 bands and the last 26 bands are noisy (Figure S1), so only 211 bands from 20 to 230 are used for subsequent analysis.

#### 2.3.3. Data Processing and Modelling

PLS is one of the most widely used regression modeling method in spectral data analysis since it can efficiently and reliably process complex spectral data [23]. In the PLSR model, the principal components of the matrix X and the matrix Y are decomposed in order to extract the most comprehensive variables with respect to the dependent variables and maximize the correlation between the principal component and the concentration, which overcomes the negative effects of the multiple correlation of variables and further improves the reliability of the model [24]. The mathematical details of PLS description can be found in the reference by Wold et al. [25] In this work, PLSR models were used to predict the storage time of fish under room temperature, refrigeration, and freezing. PLS-DA method was used as the supervised classification method to classify fresh, refrigerated, and frozen–thawed fish.

HSI has a high dimensionality with collinearity and redundancy among contiguous wavelengths [26]. Some congruent wavelengths are related to the similar constituents while some bands may contain irrelevant information or noise. Therefore, wavelength selection should be conducted to optimize data analysis and save time to compute. In this study, CARS was applied to simplify and optimize regression or classification models. The competitive adaptive weighted algorithm method, which imitates the evolution of the “survival of the fittest” principle, phases out of the invariable wavelength [27,28]. The specific steps of the algorithm are as follows:

(1) Use Monte Carlo method to collect samples *n* times. Each time a certain proportion of samples are randomly selected from the sample set as the calibration set.
(2) Establish the PLS regression model by using the extracted spectral matrix X (*n* × *m*) and the concentration matrix Y (*n* × 1).
(3) Use the exponentially decreasing function (EDF) to delete the wavelength points with small absolute value of regression coefficient. Collect samples for i times and determine the retention rate of wavelength points where a and k are constants according to the EDF calculation formula. It is calculated as follows:

(2) $$a={\left(\frac{m}{2}\right)}^{\frac{1}{N-1}}$$

(3) $$k=\frac{\ln\left(\frac{m}{2}\right)}{N-1}$$

(4) In the process of *N* sampling, the wavelength variables with large absolute values of the PLS regression coefficients are retained, while wavelength variables with small absolute values of the regression coefficients are eliminated, and then the optimal subset of wavelength variables is selected according to the RMSECV value in the model.

**Figure 1:**
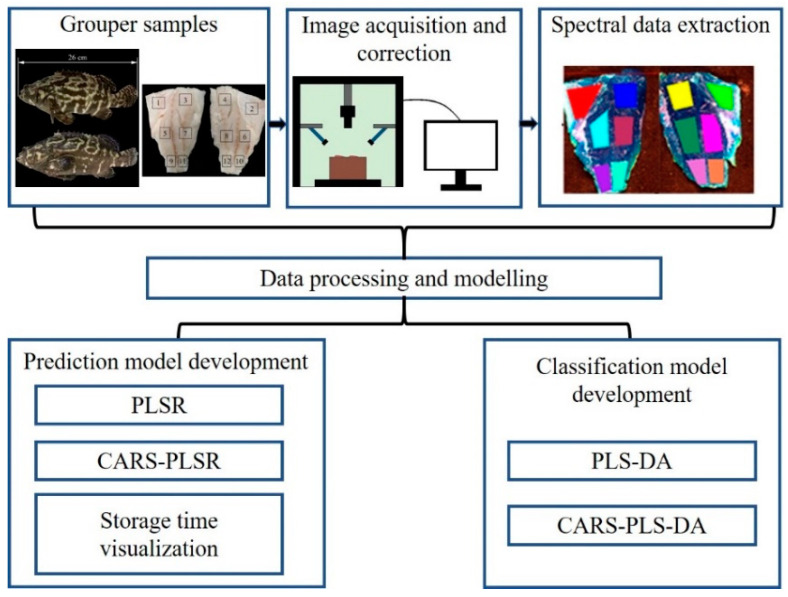
Workflow chart for data processing.

In order to visually present dynamic change of fish freshness from diverse storage time and temperature, a color hyperspectral map was generated [7]. The obtained optimal PLS models were employed on each pixel of the ROIs in the corresponding HSI, and the prediction value of each pixel based on the spectrum was calculated. With a linear color scale change, color hyperspectral images were generated according to the prediction values. In this way, it is easy to observe the change of fish freshness. All the related operations were realized by an image processing program in Matlab 2018a software (The Mathworks, Inc., Natick, MA, USA). Key steps for the data processing are summarized in Figure 1.

#### 2.3.4. Model Performance Evaluation

Determination coefficient of calibration (R_c_^2^) and prediction (R_p_^2^), root mean square errors of calibration (RMSEC), root mean square error for prediction (RMSEP), and residual predictive deviation (RPD) are the main parameters for evaluating the prediction models. Generally, a good prediction model should have high R_c_^2^ and R_p_^2^, low RMSEC and RMSEP [29]. The quality of the PLSR model can be evaluated by the RPD values, a RPD value greater than 2.0 indicates a good quantitative model, while larger than 3.0 means that the model is excellent [30]. The performance of the PLS-DA models for discrimination of fresh, refrigerated, and frozen–thawed fish was determined by the classification accuracy both in the calibration and prediction sets.

## 3. Results and Discussion

### 3.1. Analysis of Different Parts of Fish

**Figure 2:**
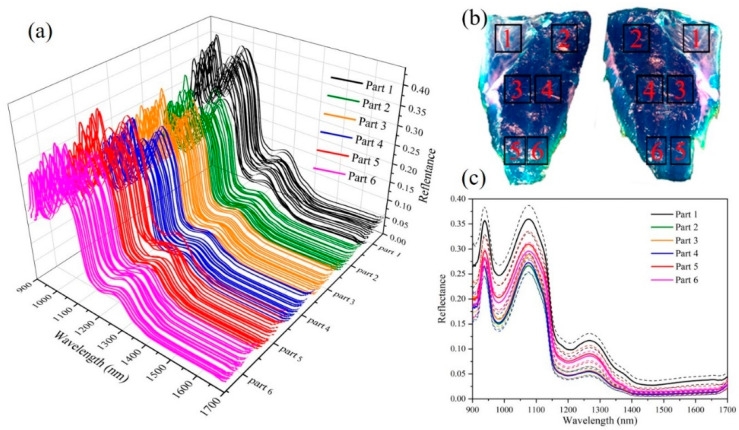
Hyperspectral data of different parts of 22 fresh fish: (**a**) Raw spectra of 6 parts of 22 fresh fish; (**b**) 6 parts of fish sample; (**c**) mean and standard deviation spectra of 6 parts.

The raw reflectance spectra within the 6 parts (Figure 2b) of 22 fresh fish are presented in Figure 2a; these parts of fish are collected from back, belly, and tail. In order to clearly show the difference in the spectra of different parts of fish, the mean and standard deviation spectra of six parts are displayed in Figure 2c. Some broadband peaks occurring in the near infrared region can be well explained by the overtone and combination vibrations of the molecular chemical bonds, such as O-H, C-H, and N-H [31]. The presence of water in the pearl gentian grouper showed two absorption bands at 980 nm and 1450 nm due to O-H stretching second and first overtones [32] in Figure 2c. Besides, one absorbance peak located at 930 nm corresponded to the third overtone C–H stretching in the methylene group of fat [33], the other absorption peak around 1220 nm was ascribed to the C-H stretch second overtone of fat [34]. It was also observed that the spectral reflectance curves of samples were quite smooth with similar trends across the whole tested wavelength region. However, a remarkable difference in the magnitudes of spectral reflectance value among different parts of fish was observed. The different reflectance spectra of different parts are mainly due to the difference in fat content. The lower the fat content, the higher the absorbance and the lower the reflectivity. Generally, the natural distributional pattern of fat, increasing from back to belly, and from tail to head [35]. Parts 2, 3, and 4 are mainly located on the back and have low fat content, so the reflectance spectrum value is low. While site 1 is located in the belly, the high fat content results in a large reflection spectrum amplitude. However, fat content generally decreases from skin side to inside [35], the 5 and 6 parts of the tail may be because the fish is very thin and very close to the skin, resulting in higher fat content.

### 3.2. Classification Model Development

**Figure 3:**
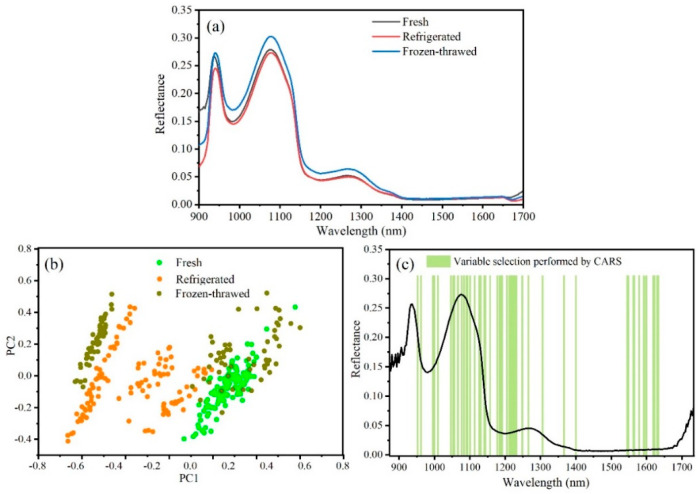
Classification model for fresh, refrigerated, and frozen–thawed fish: (**a**) Average spectra of fresh, refrigerated, and frozen–thawed fish; (**b**) classification results performed by principal component analysis (PCA) model; (**c**) variable selection performed by competitive adaptive reweighted sampling (CARS).

In order to clearly show the differences in the spectra of fresh, refrigerated, and frozen–thawed fish, the spectra of all samples under each condition were averaged and placed in the same coordinate axis, as shown in Figure 3a. It was obvious that frozen–thawed fish exhibited higher reflectance values in the whole wavelength range, and the reflectance curves of fresh and refrigerated fish were approximate. This was caused by the changing of the major chemical compositions in fish during the freezing process [36]. During the freezing–thawing process of fish, the formation and growth of ice crystals can cause tissue damage, texture deterioration, cell rupture, and organelle leakage; trimethylamine oxide was decomposed into trimethylamine, dimethylamine, and formaldehyde. The interaction between formaldehyde and fish protein not only accelerated protein denaturation, but also leaded to the deterioration of texture. The denaturation of protein will further reduce the water capacity of the protein. In summary, the water in the fish was lost during the freezing–thawing process, and the water content of the thawed fish was reduced. Meanwhile, water was the most important component of the fish meat, so the reflectance spectrum values of the frozen–thawed fish meat were higher. However, the water loss of refrigerated fish was less, and the ingredient content was very close to the fresh fish, so the reflectance spectrum value was also close.

Figure 3b shows the results of principal component analysis (PCA) on the fresh, refrigerated, and frozen–thawed fish. It can be seen that fresh and refrigerated sample points were generally clustered into two groups based on their first two orthogonal PCs. However, the effect of frozen–thawed sample classification was not good, and there was a serious overlap with the previous two. Whether it was refrigerated or frozen fish, the division into different clusters was mainly caused by different days of data collection. The longer the refrigeration or freezing time, the more obvious the difference from fresh fish. For refrigerated fish, the samples before the fifth day were basically distinguished from the fresh fish, and they gradually became distinct after the fifth day. For frozen fish, there was a clear overlap between the samples before the fifth day and the fresh fish, while the samples after the 10th day had been clearly distinguished. In addition, the optimal variables extracted by CARS were used to build the PCA model (Figure S2), but it was found that the effect was not as good as the full-spectrum model.

PLS-DA and CARS-PLS-DA algorithms (Figure 3c) were used to classify the fish under different storage conditions. The classification results are displayed in Table 1. As can be seen, calibration set and prediction set were allocated according to 3:1. The results showed that PLS-DA and CARS-PLS-DA algorithms could classify fresh, refrigerated, and frozen–thawed fish well, but PLS models was better overall. The PLS-DA model had 211 variables. For PLS-DA model developed with spectra of fresh fish, all fresh fish were identified correctly. For refrigerated fish and frozen–thawed fish, there was one sample misclassified, respectively, with accuracy of 96.43%. The CARS-PLS-DA model selected 48 variables, far less than the PLS-DA model. The same results were observed when CARS-PLS-DA models were developed with spectra from fresh and refrigerated fish, with accuracy of 96.43%. However, the accuracy of frozen–thawed fish was only 89.29%, with three samples misclassified as refrigerated fish. Although the classification accuracy of the PLS-DA models was reduced, the efficiency of the programs was greatly improved.

### 3.3. Prediction Model Development

**Figure 4:**
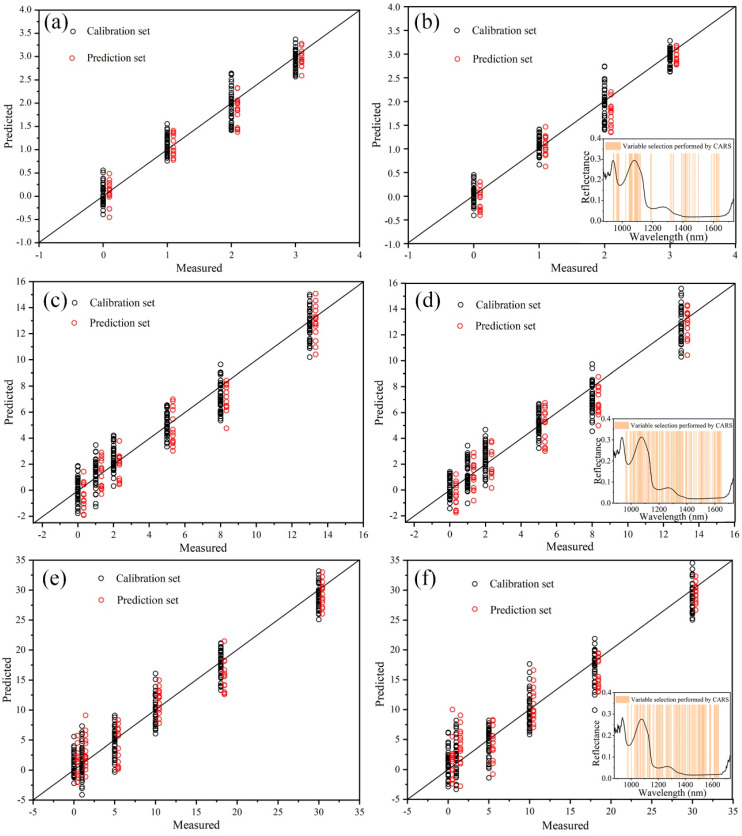
PLS regression (PLSR) and competitive adaptive reweighted sampling-PLSR (CARS-PLSR) models of storage time under different storage conditions: (**a**,**b**): Room temperature; (**c**,**d**) refrigeration; (**e**,**f**) freeze.

Figure 4 shows the PLSR and CARS-PLSR models of storage time under three storage conditions using the entire spectrum. Figure 4a,c,d shows scatter distribution diagrams of storage time predicted by PLSR model for prediction set samples. The abscissa is the real refrigeration time, and the ordinate is the predicted time. The samples were distributed around the ideal prediction line and were relatively close to the straight line, indicating that the hyperspectral and PLSR algorithm could accurately predict the storage time of fish. 

To make the HSI system suitable for implementation in pearl gentian grouper processing lines, it is important to reduce the high dimensionality of the hyperspectral cubes and to build a simplified spectral model [37]. The above analysis based on the full spectral range did not take into account that some spectral wavelengths might not contain any useful information with regard to the storage time of the samples [32]. Therefore, the significant variables/wavelengths reflecting the characteristics of spectra for predicting storage time were collected using the CARS method. As a result, new reduced spectral matrices were created and then used to replace the full wavelengths spectra for building new calibration models to determine storage time under different storage conditions. The wavelengths obtained of fish under different storage conditions (Figure 4b,d,f) were recognized as the important variables for further predicting storage time in the fish. In combination with Table 2, we can see that the number of variables extracted from the spectrum of fish stored at room temperature, refrigeration, and freezing conditions were 49, 119, and 99, respectively. Figure 4b,d,f shows scatter distribution diagrams of storage time predicted by CARS-PLSR model for prediction set samples. The abscissa is the real refrigeration time, and the ordinate is the predicted time. Visually, it can be seen that the prediction results of samples stored at room temperature, refrigerator and freezer were sequentially worsening.

The statistical results of calibration and prediction models are presented in Table 2. For fish stored at room temperature (R_p_^2^ = 0.948, RMSEP = 0.255, RPD = 4.380) and refrigerator (R_p_^2^ = 0.9319, RMSEP = 1.188, RPD = 3.857), the model after the feature band extraction had a better prediction effect than the PLS regression model based on the full band. However, PLSR prediction results of fish stored at freezer were more satisfactory than CARS-PLSR, for which R_p_^2^ was 0.250 (RMSEP = 2.910, RPD = 3.469).

In addition, we screened out the same characteristic bands of fish under room temperature, refrigerated, and freezing conditions (Figure S3), and used these characteristics to build the model; the results obtained are shown in Table S1, but the prediction results under the three conditions were not as good as the previous models.

### 3.4. Storage Time Visualization

**Figure 5:**
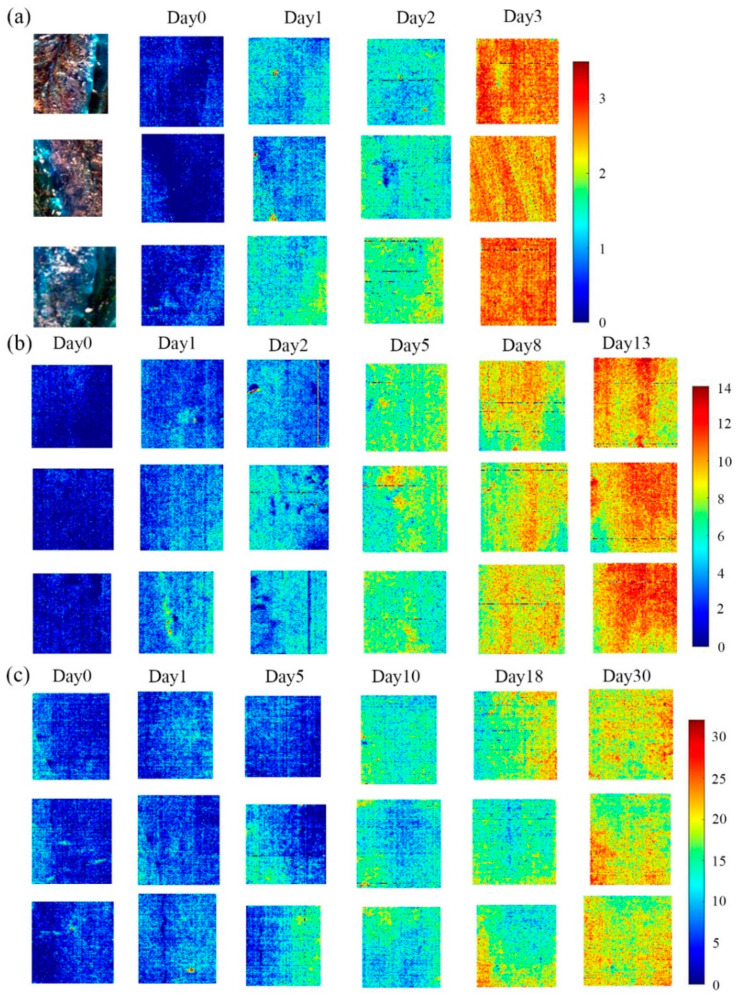
Visualization of storage time under different storage conditions: (**a**) Room temperature; (**b**) refrigeration; (**c**) freeze.

It can be seen from Table 2, for fish stored at room temperature and refrigerator, the prediction results of PLSR and CARS-PLSR were extremely close. However, for fish in frozen condition, PLSR obtained relatively obvious better prediction results. In comprehensive consideration, PLSR was selected as the optimal model. The optimal model was employed on each pixel of HSI, and the prediction values were calculated and presented in corresponding color. The predicted time value of each pixel was then mapped with a linear color scale, where the different time values from small to large were shown in different colors from blue to red. The color images visually showed the storage time change of fish under different storage conditions in Figure 5. Generally speaking, pixels with similar spectral characteristics would have similar predicted value of the storage time, leading to a similar scale in the generated color images.

As can be seen from the linear color bar in the lower right corner of the figure, dark blue represents the shortest refrigeration time of 0 days, and dark red represents the longest storage time, 3 days, 12 days, and 30 days, respectively. The gradient from dark blue to dark red represents the extension of storage time, and different colors represent different days. In Figure 5a, from top to bottom, there were three samples stored for 0, 1, 2, and 3 days at a room temperature of 10–25 °C. The color gradually changed from dark blue to red. In Figure 5b, from top to bottom, samples were refrigerated for 0, 1, 2, 5, 8, and 13 days. The color gradually changed from dark blue to red. Figure 5c shows samples from 0, 1, 5, 10, 18, and 30 days later under freezing conditions from top to bottom. The color gradually changed from dark blue to light red. In brief, the actual storage time under the three storage conditions were basically consistent with the predicted refrigeration time corresponding to the color in the figure, indicating that the PLSR models established by the average spectrum can accurately predict the storage time of each pixel on the unknown samples.

It can be seen from Figure 5a that the yellow and orange pixels of fish under the storage condition of 10–25 °C at room temperature increased significantly after three days, indicating that the freshness of fish were rapidly decreasing. The fish images on the third day were almost all red pixels, indicating that the fish had basically deteriorated at that time.

For fish under refrigerated conditions at 4–7 °C (Figure 5b), there were mostly blue pixels in the images within 1–2 days, yellow and orange pixels began to increase, and the freshness of the fish were decreasing. The yellow and orange pixels in the images on the fifth to eighth day increased significantly, and the freshness of the fish at this stage decreased rapidly. The fish images on the 13th day were basically full of red pixels, and at this time the fish were close to deterioration. Compared with fish under room temperature, the deterioration rate of fish under refrigeration was significantly slower.

For fish in frozen condition (Figure 5c), there were mostly blue and green pixels in the images of 0–5 days, and the color change speed was relatively slow over time, indicating that the freshness of the fish decreased slowly at this stage. In the images from 10 to 30 days, the yellow and orange pixels only began to increase, indicating that the freshness of the fish decreased to a large extent. However, the decline rate was still very slow, much slower than fish under refrigerated conditions.

In addition, due to the natural heterogeneity of fish tissue, the physical and chemical properties of each pixel were not completely consistent, and the predicted value of storage time were not completely consistent [38]. Therefore, although the storage time of the same sample was the same, the color on the image was not completely uniform, but the freshness of the sample can still be judged based on the color of most pixels. The bluer the color, the fresher the fish, and the redder, the less fresh the fish. Different from previous studies, this study visualized the storage time of fish under three storage conditions, visually and intuitively showing the freshness status and distribution of fish. In the actual production, processing, and sales in the future, producers can cut and grade the fish according to different freshness requirements, and at the same time, it is convenient for consumers to quickly choose and judge.

## 4. Conclusions

In this paper, HSI technology was used to study the identification of fresh, refrigerated, and frozen–thawed pearl gentian grouper, and realized the rapid and accurate prediction of storage time and distribution visualization of pearl gentian grouper under different storage conditions. First, by analyzing the average reflectance spectra of different parts of the fish sample, three parts with smaller spectral values were selected for subsequent analysis. The PLS-DA and CARS-PLS-DA classification models were used to identify the fresh, refrigerated, and frozen–thawed fish. The results showed that the PLS-DA classification results were better overall, and the accuracy of fresh, refrigerated, and frozen samples were 100%, 96.43%, and 96.43%, respectively. Additionally, the PLSR and CARS-PLSR models were developed to predict the storage time of the prediction set samples under three storage conditions. Both models have achieved high modeling and prediction accuracy. For fish under room temperature and refrigeration, the results of CARS-PLSR were better (R_p_^2^=0.948, RMSEP = 0.255) the R_p_^2^ were 0.948 and 0.9319, respectively, the RMSEP were 0.255 and 1.188, respectively, and the RPD were 4.380 and 3.857, respectively. For fish under freezing conditions, PLSR performed the better results, the R_p_^2^ was 0.9250, the RMSEP was 2.910, and the RPD was 3.469. Finally, the PLSR models were used to predict the storage time of each pixel on the sample images of the prediction set, combined with the image programming technology of MATLAB software, to realize the visualization of the predicted storage time in the form of a pseudo-color map. This research laid the foundation for the widespread application of HSI technology in the field of aquatic product processing and the automation of aquatic product processing in the future.

## Figures and Tables

**Table 1:** Classification results based on partial least square- discrimination analysis (PLS-DA) and competitive adaptive reweighted sampling-PLS-DA (CARS-PLS-DA) models.

Model	Variable Number	Group	Calibration Set				Prediction Set			
			1	2	3	Accuracy/%	1	2	3	Accuracy/%
PLS-DA	211	1	82	2	0	97.62	28	0	0	100
		2	0	83	1	98.81	0	27	1	96.43
		3	0	3	81	96.43	0	1	27	96.43
CARS-PLS-DA	48	1	81	3	0	96.43	27	1	0	96.43
		2	1	81	2	96.43	1	27	0	96.43
		3	0	14	70	83.33	0	3	25	89.29

Group 1: Fresh; Group 2: Refrigerated; Group 3: Frozen-thawed.

**Table 2:** Prediction results based on PLS regression (PLSR) and competitive adaptive reweighted sampling-PLSR (CARS-PLSR) models.

Condition	Model	Variable Number	Calibration Set			Prediction Set			
			Number	R_c_^2^	RMSEC	Number	R_p_^2^	RMSEP	RPD
room temperature	PLSR	211	162	0.9464	0.259	54	0.9448	0.263	4.144
	CARS-PLSR	49		0.9557	0.235		0.948	0.255	4.380
refrigeration	PLSR	211	243	0.9426	1.081	81	0.9304	1.200	3.865
	CARS-PLSR	119		0.9370	1.133		0.9319	1.188	3.857
freeze	PLSR	211	243	0.9500	2.353	81	0.9250	2.910	3.469
	CARS-PLSR	99		0.9324	2.735		0.9152	3.094	3.222

## Data Availability

The data presented in this study are available on request from the corresponding author.

## Supplementary Materials

Supplementary materials can be found at https://www.mdpi.com/1424-8220/21/2/583/s1. Figure S1: Full band spectrum of fish sample. Figure S2. Classification results performed by principal component analysis (CARS-PCA) model. Figure S3. The same characteristic bands of fish under room temperature, refrigerated and freezing conditions. Table S1. Prediction results based on the common characteristic bands performed by PLS regression (PLSR).
