# Supplementary material for: Hyperspectral Imaging (HSI) Technology for the Non-Destructive Freshness Assessment of Pearl Gentian Grouper under Different Storage Conditions
Source: Sensors (Basel). 2021 Jan 15;21(2):583. doi: 10.3390/s21020583 (PMC7830432; doi:10.3390/s21020583)
Supplement: Supplementary file 1 [file sensors-21-00583-s001.pdf]

Article

Hyperspectral Imaging (HSI) Technology for the Non-Destructive Freshness Assessment of Pearl Gentian Grouper under Different Storage Conditions

Zhuoyi Chen [1,2], Qingping Wang [1,2], Hui Zhang [1,2] and Pengcheng Nie [1,2,3,*]

1 College of Biosystems Engineering and Food Science, Zhejiang University, Hangzhou 310058, China; zhuoyichen@zju.edu.cn (Z.C.); 22013049@zju.edu.cn (Q.W.); 21813051@zju.edu.cn (H.Z.);

2 Key Laboratory of Sensors Sensing, Ministry of Agriculture, Zhejiang University, Hangzhou 310058, China

3 State Key Laboratory of Modern Optical Instrumentation, Zhejiang University, Hangzhou 310058, China

* Correspondence: npc2012@zju.edu.cn; Tel.: +86-0571-88982456

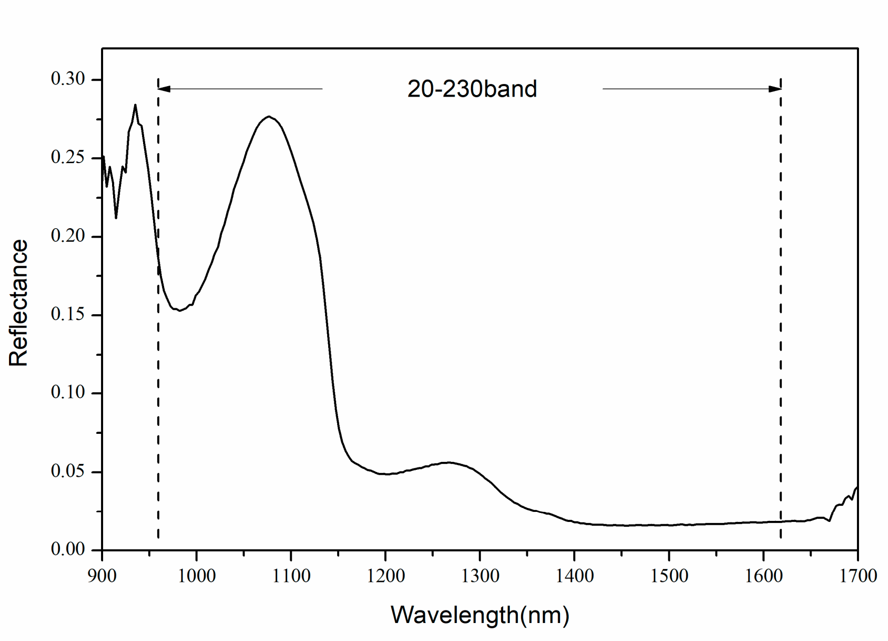

Figure S1. Full band spectrum of fish sample.

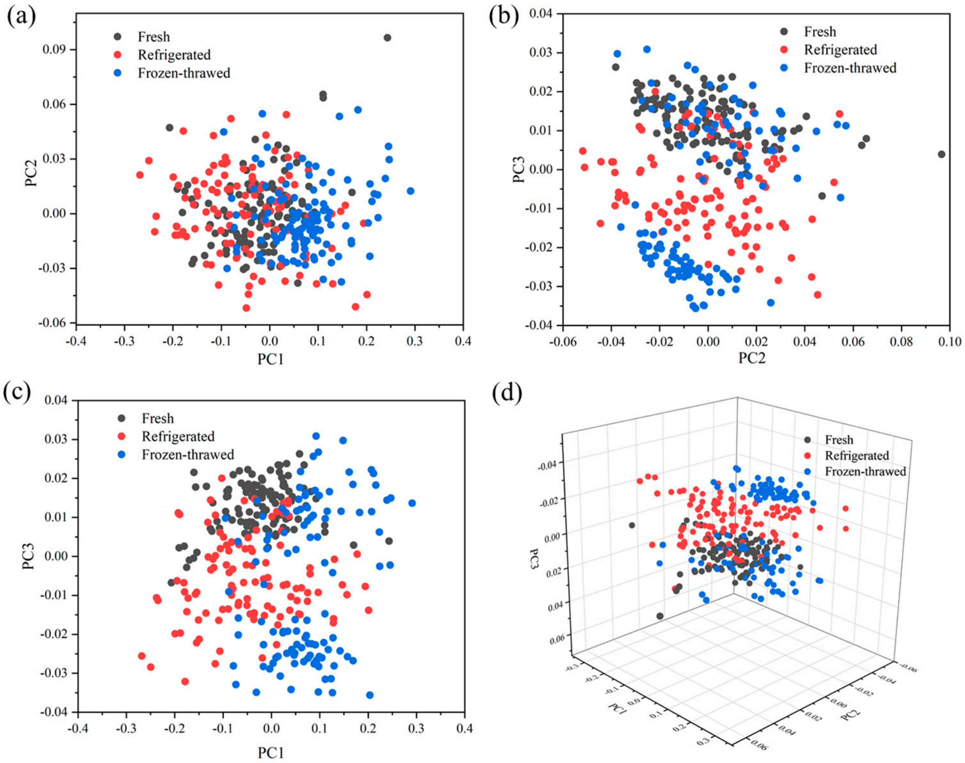

Figure S2. Classification results performed by principal component analysis (CARS-PCA) model.

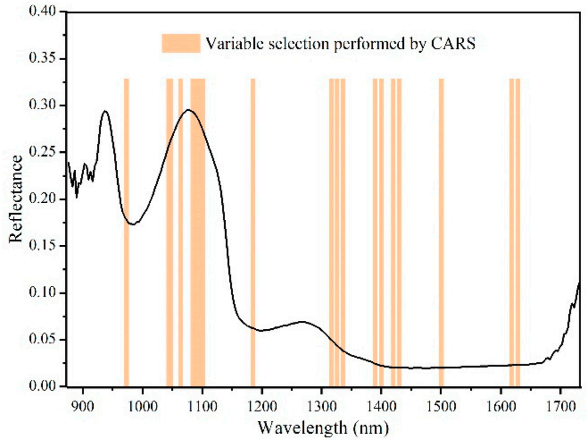

Figure 3. The common characteristic bands of fish under room temperature, refrigerated and freezing conditions.

Table S1. Prediction results based on the common characteristic bands performed by PLS regression (PLSR).

Condition	Model	Variable number	calibration set			Prediction set			
			Number	R_c^2	RMSEC	Number	R_p^2	RMSEP	RPD
room temperature	PLSR	19	162	0.9291	0.2977	54	0.8771	0.3919	2.764
refrigeration			243	0.6904	2.5124	81	0.6921	2.5256	1.545
freeze			243	0.5549	7.0183	81	0.5231	7.3363	1.009
